# Healthcare professionals’ knowledge, attitudes, and practices regarding China’s National Centralized Drug Procurement policy: a cross-sectional study in Shaanxi Province

**DOI:** 10.3389/fpubh.2025.1616534

**Published:** 2025-08-20

**Authors:** Qian Shen, Limin Kang, Dan Ye, Youjia Li, Kanghuai Zhang

**Affiliations:** ^1^Department of Pharmacy, The Second Affiliated Hospital of Xi’an Jiaotong University, Xi’an, China; ^2^Department of Pharmacy, Xi’an No.3 Hospital, The Affiliated Hospital of Northwest University, Xi’an, China

**Keywords:** NCDP policy, KAP, healthcare professionals, centrally procured drugs, generic consistency evaluation

## Abstract

**Background:**

Since 2019, the Chinese government has been fully implementing the National Centralized Drug Procurement (NCDP) policy, with the core objective of reducing drug costs and alleviating the medical burden on patients. As the practitioners of this policy, healthcare professionals (HCPs) play a crucial role in its implementation. Therefore, it is necessary to evaluate their knowledge, attitudes, and practices (KAP) regarding the implementation of the (NCDP) policy and the factors influencing these aspects.

**Methods:**

We conducted a cross-sectional online survey on HCPs from public hospitals in Shaanxi Province from 2022 August to September. A structured self-administered questionnaire was used to collect data on demographics, knowledge of NCDP, attitude toward NCDP policy, and practice of policy implementation. KAP adequacy was dichotomized at the 70% cut-off (Knowledge: adequate vs. inadequate, Attitude: positive vs. negative; Practice: good vs. poor). All the variables were descriptively analyzed, and multivariate logistics regression analysis was used to explore the factors associated with KAP level.

**Results:**

A total of 1,257 valid responses were received. Of these, 58.4% demonstrated adequate knowledge, 63.2% held positive attitudes, and 35.2% exhibited good practices. Significant occupational disparities emerged, with pharmacists outperforming physicians across all KAP domains (*p* < 0.01). Multivariate logistic regression analysis showed that professional title, being a pharmacist, and attending the training were independently associated with adequate knowledge. Positive attitudes were significantly associated with 11–20 years of working experience, having a bachelor’s degree, being a pharmacist, and attending training. Proactive practice was associated with longer working years, being a pharmacist, and training attendance. The main concerns of HCPs regarding the NCDP policy were guaranteeing medicine quality (93.2%), strengthening clinical outcomes research (80.0%), and strengthening monitoring of adverse reactions (79.6%).

**Conclusion:**

Our study found that HCPs in Shaanxi Province possessed a moderate level of knowledge and generally positive attitudes toward the NCDP policy, yet demonstrate room for enhanced practice. Pharmacists play a pivotal role in policy implementation. To strengthen HCPs’ policy endorsement and ensure faithful implementation, targeted training on therapeutic effectiveness and safety evaluation is essential, as it can alleviate clinical concerns and bridge the gap from evidence to actionable competence. Future research should concentrate on generating high-quality clinical evidence, analyzing long-term policy impacts, identifying implementation barriers, and designing educational interventions to bolster HCPs’ knowledge and positive attitudes regarding the NCDP policy.

## 1 Background

Global healthcare costs are rising rapidly. Notably, the WHO reported $9 trillion in spending in 2020, accounting for 11% of the world’s GDP, and this trend is expected to continue (1). Drug expenditure has always been a significant component of national and individual medical costs (2). Moreover, drug expenditure accounts for 17% of total health spending in higher-income countries, while in lower-middle-income countries, the proportion can be as high as 70% (3, 4). In response to the growing burden of healthcare costs, governments and health organizations worldwide have implemented various strategies to control healthcare costs, including measures to manage drug expenditure (5–7). Empirical studies also repeatedly highlight the significant effectiveness of bulk purchasing in reducing the costs of pharmaceuticals that are successfully incorporated into procurement contracts (8, 9). Many countries, such as India, Brazil, and Denmark, have successfully implemented centralized drug procurement strategies. As such, they achieve notable reductions in drug prices and significant savings in pharmaceutical costs (10, 11). This global trend is evident in China, where pharmaceutical expenditure reached US$169 billion in 2021. In response to the ongoing challenges of limited healthcare accessibility and medication adherence, China introduced the National Centralized Drug Procurement (NCDP) policy in January 2019. This aims to reduce the price of drugs further, alleviate the medication burden for patients, improve drug availability, and deepen public hospital reform.

The first round of the NCDP policy, also known as the “4 + 7” policy, was so named because 11 cities were selected as pilot cities, including 4 municipalities (Beijing, Tianjin, Shanghai, Chongqing) and 7 sub-provincial cities (Shenyang, Dalian, Xiamen, Guangzhou, Shenzhen, Chengdu, Xi’an). This policy implements “volume-based procurement,” meaning that the tenderer (the government) specifies the procurement volume (60–70% of the medicine consumption of all public medical institutions in pilot cities in the previous year) during the bidding. The bidders (pharmaceutical companies) then quote prices according to the specific volume. Furthermore, the pharmaceutical company with the lowest price for each medicine wins the bid. To ensure that domestically produced generic drugs are therapeutically equivalent to their corresponding brand-name versions in terms of quality, safety, and efficacy, the NCDP policy mandates that all centrally purchased generic drugs pass the Generic Consistency Evaluation (GCE), which requires rigorous bioequivalence testing and pharmaceutical quality standardization (12). In order to be bioequivalent, National Medical Products Administration (NMPA) required that the 90%CI of the geometric mean ratio for main pharmacokinetic parameters, the peak concentration and the area under concentration time curve of the product fall entirely within the range of 80.00–125.00% (13). As of 2024, GCE implementation has enabled 10 rounds of centralized procurement, covering 435 drugs that met these equivalence standards (14). Currently, these drugs cover multiple therapeutic areas, such as medications for chronic diseases like metformin (for diabetes) and amlodipine (for hypertension), antitumor drugs including gefitinib (targeted therapy) and oxaliplatin (a chemotherapeutic agent), and antibiotics like cefdinir and meropenem. The policy has demonstrably reshaped China’s pharmaceutical landscape, yielding significant outcomes across multiple dimensions. Empirical evidence indicated: ① a substantial shift toward generic drugs, with their procurement volume share rising from 60.73 to 77.80%, displacing 17.08% of originator drugs (13); ② improved clinical rational drug use, driven by volume guarantees, performance evaluations promoting quality generics (especially GCE-certified in primary care), and financial disincentives reducing high-cost drug overprescribing (12, 13); ③ reduced patient drug burden, exemplified by a 20.75% decrease in inpatient drug expenditures for conditions like lung cancer (15); and ④ enhanced health insurance efficiency. As of the end of 2021, the policy has saved over 260 billion CNY (approximately US$36.3 billion) (12). NCDP policy has significantly reduced healthcare costs and improved drug accessibility. However, its real-world effectiveness ultimately depends on the active participation and precise execution by frontline healthcare professionals (HCPs), including clinicians (physicians, pharmacists, nurses) directly handling medications and administrators responsible for policy operationalization. In this context, HCPs serve as policy implementers and dynamic interpreters. They are also the main executors of the policy, particularly physicians, who play a key role in generic drug use through their central position as decision-makers for pharmacotherapy (16). The policy’s guiding principles encourage physicians to prioritize centrally procured medicines, limiting their prescription options for non-centrally procured drugs and indirectly affecting patients’ medication adherence. As an integral part of the healthcare system, pharmacists are crucial in promoting the generic drug policy. They collaborate closely with physicians to provide professional advice on generic drug selection, dosage, interactions, and side effects. Additionally, they educate and counsel patients during medication dispensing, thereby enhancing physicians’ acceptance of centrally procured drugs (17). To ensure the effective execution of the policy, relevant departments have introduced implementation opinions and measures to deepen the reform of centralized drug procurement and to establish a national public drug procurement market (18). Therefore, understanding the perceptions and practices of healthcare professionals regarding the NCDP is crucial for optimizing drug usage and ensuring the success of the policy.

Knowledge, attitude, and practice (KAP) surveys serve as an effective instrument for exploring health behaviors and the ways people seek healthcare (19). Previous studies (17, 20) have evaluated the KAP of generic substitution in China among HCPs in different regions. Moreover, researches (21, 22) have shown that the prices of drugs selected in centralized procurement across China are inversely related to economic development. Economically advanced eastern regions, which enjoy more convenient transportation, denser populations, and higher medication demand, are typically more likely to be chosen by enterprises offering lower prices. In contrast, less economically developed areas with lower drug demand are more likely to be selected by higher-priced enterprises. Given that Shaanxi Province, located in northwest China, has a diverse economic profile with both developed and developing regions, assessing the KAP of HCPs in Shaanxi regarding the centralized drug procurement policy is essential.

## 2 Methods

### 2.1 Study design and setting

A descriptive cross-sectional survey was conducted among HCPs in public health institutions from August 1st to September 1st, 2022, in Shaanxi Province, located in northwestern China, and ranks first in GDP (Gross Domestic Product) among the five northwest provinces of China. Additionally, it is the starting point of the Silk Road and an important center for culture and tourism (23). Shaanxi consists of 11 cities and 69 counties, with a population of 39.52 million and an area of 205,800 square kilometers (24). By the end of 2022, Shaanxi had 34,779 health institutions. Among them, there are 1,280 hospitals, including 443 public hospitals. The province had 289,600 hospital beds and 378,200 healthcare professionals, including physicians, nurses, pharmacists, laboratory officer, radiology officer and dentist. (25).

### 2.2 Sample size calculation

The sample size was calculated using Calculator.net.^1^ We ran a statistical power analysis for sample size calculation with a population percentage of 50%, a margin of error of 0.04, and a confidence level of 99%. The required sample size was 1,038. The theoretical sample size was calculated to be 1,298, including an additional 25% to account for potential subject loss.

### 2.3 Instrument

A structured self-administered questionnaire was used to collect the data. The investigators developed the questionnaire in the national language (Chinese) after conducting an extensive literature review (26–28) and discussion with two experts: a senior pharmaceutical expert with 30 years of hospital pharmacy experience (ensuring policy terminology accuracy) and a hospital procurement officer (verifying operational feasibility of workflow-related items). Following finalization of the preliminary questionnaire draft, two domain experts from a provincial tertiary teaching hospital conducted peer reviews to evaluate content validity and readability. The pharmacy administration expert (Prof. KHZ) proposed incorporating items addressing HCPs’ key concerns regarding NCDP implementation, while the chief physician (Dr. XQY) recommended augmenting the introductory section with a standardized definition of GCE.

Furthermore, the questionnaire was pilot-tested among 30 HCPs of our hospital (10 physicians, 12 pharmacists, and 8 nurses). The Cronbach’s alpha was calculated as 0.81 (knowledge), 0.86 (attitude), and 0.82 (practice), respectively, indicating acceptable internal consistency. Notably, Kaiser-Meyer-Olkin and Bartlett’s sphericity test revealed the KMO value was 0.878, showing good validity.

The questionnaire consisted of 33 items divided into four sections (see Supplementary Appendix): (1) demographic characteristics, for example gender, age, working years, professional title, education level, occupation, hospital category, and attending training(related to centralized procurement policies) or not; (2) knowledge of NCDP (7 items) about generic drug consistency evaluation, volume-based purchasing policy, the efficiency and safety of branded and generic drugs and impact on medical insurance fund expenditure; (3) attitude toward NCDP policy (5 items), about generic drug substitution, clinically rational drug use, medication burden, and efficiency of insurance fund, etc.; (4) practice of policy implementation (3 items), such as the possibility of using centralized purchased drugs by themselves or their families. The knowledge section was assessed through the respondents’ answers to the questions with “Yes” or “No”; “Yes” received 1 point while “No” received 0. In the attitude and practice section, a 5-point Likert scale either of agreement (strongly disagree, disagree, neither, agree, strongly agree) or frequency (never, occasionally, sometimes, often, always) was used. We categorized each KAP domain using a 70% competency threshold (29). Specifically, knowledge scores of ≥5 were classified as “adequate,” while those <5 were deemed “inadequate.” For attitude scores, ≥18 was labeled as “positive,” and <18 was considered “negative.” Practice scores were rated as “good” if ≥11 and “poor” if <11. The pilot study results were not included in the final results. Questionnaires with obvious logical errors or a pattern of selecting the same options for all items were considered invalid and excluded from the analysis.

### 2.4 Data collection procedure

The inclusion criteria were HCPs working at public hospitals in Shaanxi Province at the time the survey conducted. The questionnaire using screeners that automatically excluded any respondents whose filling time was less than 100 s or whose work location was not in Shaanxi Province.

A convenience and snowball sampling strategy was used to enroll potential participants. First, the questionnaire was created and published on a professional platform named “WenJuanXing” for data collection. The questionnaire was made into a QR code and sent to the pharmacy department director of our unit using WeChat as the carrier. Then, the department director sent the QR code to WeChat groups of various pharmaceutical specialized committees and invited the directors of the clinic department of hospitals to be the first batch of research objects. Finally, the first batch of research subjects would publish the questionnaire in the workgroup of their department, invite the staff of their department as the second batch of research subjects to complete the questionnaire, and encourage their colleagues to send the questionnaire to their WeChat Moment and invite their peers to fill in the questionnaire.

### 2.5 Statistical analysis

Data accuracy and completeness were verified prior to analysis using SPSS 25.0 (IBM Corp.). Descriptive statistics presented continuous variables as mean±SD and categorical variables as frequency (%). Pearson correlation analyses were performed to examine linear relationships between continuous knowledge, attitude, and practice scores. Potential factors associated with KAP levels were initially screened using Chi-square tests between all baseline characteristics and the binary KAP outcome. Variables with *p* < 0.1 in univariate analyses were entered into multivariable logistic regression to identify independent factors influencing KAP levels. A two-sided *p* < 0.05 was considered statistically significant.

## 3 Results

### 3.1 Baseline characteristics

In total, 1,283 responses were received. A total of 26 responses were excluded due to logical inconsistencies (*n* = 2, one with age >100 years, one with work experience exceeding biological age), uniform responses across all attitude and practice items (*n* = 5), or completion time <100 s (*n* = 19). After removing non-qualifying responses, the final sample size was 1,257.

The average age of the respondents was 39.27 years (SD = 9.57, range = 19–68); the majority of the respondents were female (768, 61.1%) and bachelor (700, 55.7%) from tertiary hospitals (822, 65.4%). Most respondents were pharmacists (585, 46.5%) and physicians (479, 38.1%). A description of demographic data is given in Table 1.

**Table 1:** Socio-demographic characteristics of healthcare professionals (HCPs).

Variables	Number (*N* = 1,257)	Percentage (%)
Gender		
Male	489	38.9
Female	768	61.1
Age(years)		
<30	200	15.9
30–39	486	38.7
40–49	343	27.3
≥50	228	18.1
Years of working		
≤5	263	20.9
6–10	298	23.7
11–20	316	25.1
>20	380	30.2
Professional title		
Junior	428	34.0
Intermediate	447	35.6
Senior	382	30.4
Education level		
Technical secondary school and junior college	143	11.4
Bachelor	700	55.7
Master or above	414	32.9
Occupation		
Physician	479	38.1
Pharmacist	585	46.5
Others	193	15.4
Hospital category		
Tertiary hospitals	822	65.4
Secondary hospitals	435	34.6
Attending training		
Yes	680	54.1
No	577	45.9

### 3.2 Knowledge, attitude, and practice scores

#### 3.2.1 Knowledge score

The respondents were asked seven questions about NCDP policy. The results showed that the mean score was 4.66 (SD = 2.12, range 0–7) and 516 (58.4%) had adequate knowledge. Overall, 57.1% of the respondents thought that they knew about the policy. The majority (72.6%) of respondents correctly answered six out of seven questions. NCDP knowledge scores varied significantly across working years, educational level, occupation, hospital categories, and attending training or not (Table 2).

**Table 2:** Chi-square analysis of factors associated with KAP level.

Variables	Knowledge level			Attitude level			Practice level		
	Adequate/n(%)	χ^2^	*p*-value	Positive/n(%)	χ^2^	*p*-value	Good/n(%)	χ^2^	*p*-value
Gender		0.147	0.701		0.023	0.879		3.598	0.058
Male	204 (39.5)			308 (38.7)			188 (42.4)		
Female	312 (60.5)			487 (61.3)			255 (57.6)		
Age(years)		2.955	0.399		5.413	0.144		64.557	<0.01
<30	72 (14.0)			127 (16.0)			34 (7.7)		
30–39	200 (38.8)			289 (36.4)			147 (33.2)		
40–49	149 (28.9)			227 (28.6)			150 (33.9)		
≥50	95 (18.4)			152 (19.1)			112 (25.3)		
years of working		9.42	0.024		7.913	0.048		82.545	<0.01
≤5	86 (16.7)			164 (20.6)			43 (9.7)		
6–10	132 (25.6)			187 (23.5)			90 (20.3)		
11–20	136 (26.4)			184 (23.1)			119 (26.9)		
>20	162 (31.4)			226 (32.7)			191 (43.1)		
Professional title		5.408	0.067		6.025	0.049		40.568	<0.01
Junior	161 (31.2)			252 (31.7)			109 (24.6)		
Intermediate	202 (39.1)			287 (36.1)			155 (35.0)		
Senior	153 (29.7)			256 (32.2)			179 (40.4)		
Education level		24.923	<0.01		8.042	0.018		13.437	<0.01
Technical secondary school and junior college	133 (25.8)			243 (30.6)			118 (26.6)		
Bachelor	307 (59.5)			466 (58.6)			265 (59.8)		
Master or above	76 (14.7)			86 (10.8)			60 (13.5)		
Occupation		90.663	<0.01		122.711	<0.01		140.743	<0.01
Physician	132 (25.6)			271 (34.1)			96 (21.7)		
Pharmacist	322 (62.4)			454 (57.1)			306 (69.1)		
Others	62 (12.0)			70 (8.8)			41 (9.3)		
Hospital category									
Tertiary hospitals	312 (60.5)	9.396	0.002	502 (63.1)	4.835	0.028	265 (59.8)	9.393	0.002
Secondary hospitals	204 (39.5)			293 (36.9)			178 (40.2)		
Attending training		80.369	<0.01		38.066	<0.01		95.389	<0.01
Yes	357 (69.2)			483 (60.8)			322 (72.7)		
No	159 (30.8)			312 (39.2)			121 (27.4)		

#### 3.2.2 Attitude score

The results showed that the mean attitude score was 18.19 (SD = 3.99, range 5–25) and 795 (63.2%) had positive attitude. However, only about half of the respondents agreed that, the clinical efficacy (615, 48.9%) and safety (690, 54.9%) of original and generic drugs are the same theoretically. Attitude scores varied significantly across working years, professional title, educational level, occupation, hospital categories, and attending training or not (Table 2).

#### 3.2.3 Practice score

The results showed that the mean practice score was 9.25 (SD = 2.82, range 3–15) and 443 (35.2%) had good practice. Notably, 109 (8.7%) respondents reported that neither they nor their family members never use centrally procured drugs. Practice scores exhibited significant variation across all demographic characteristics, with the exception of gender (Table 2).

#### 3.2.4 The KAP scores of HCPs in different occupation

Among the HCPs, pharmacists got the highest scores across all sections. Pharmacists’ KAP scores were 5.51 ± 1.56 for knowledge, 19.46 ± 3.25 for attitude, and 10.37 ± 2.61 for practice. In contrast, physician KAP scores were 3.95 ± 2.14 for knowledge, 17.64 ± 3.90 for attitude, and 8.33 ± 2.54 for practice. For the others, KAP scores were 3.80 ± 2.53 for knowledge, 15.72 ± 4.67 for attitude, and 8.15 ± 2.81 for practice.

### 3.3 Pearson correlations of KAP scores

Pearson correlation analysis (Table 3) revealed that all KAP scores were statistically significant. Specifically, knowledge scores showed a weak but significant correlation with attitude scores (*r* = 0.401, *p* < 0.01) and practice scores (*r* = 0.365, *p* < 0.01). This suggested that higher knowledge levels are associated with more positive attitudes and better practices, although the relationships are not particularly strong. Furthermore, the correlation between attitude and practice scores was also significant (*r* = 0.359, *p* < 0.01), indicating that positive attitudes are moderately associated with better practices.

**Table 3:** Correlation coefficients between KAP scores.

Item	Knowledge	Attitude	Practice
Knowledge	1		
Attitude	0.401	1	
Practice	0.365^a^	0.359^a^	1

^a^Indicates that the correlation is significant at *p* < 0.05 (two-tailed).

### 3.4 Attitudes and practices of diverse healthcare professionals toward NCDP policy

Significant occupational disparities were observed in attitudes and practices toward the NCDP policy. Pharmacists generally demonstrated a positive attitude toward the NCDP policy, with over 84% affirming its effectiveness in reducing patient burden (88.2% agree/strongly agree) and improving fund efficiency (84.0%). They also showed proactive implementation through frequent catalog tracking (56.2%), patient education (48.6%), and personal drug use (58.9%). Physicians showed economically aligned but operationally constrained profiles, supporting patient burden reduction (74.3%) but resisting clinical behavior regulation (37.1% disagreed on generic substitution), reflected in limited advocacy practices—only 25.5% tracked catalogs frequently and 28.8% routinely educated patients(see Table 4).

**Table 4:** Attitudes and practices of diverse healthcare professionals toward NCDP policy.

Item	Pharmacists (*n* = 584)	Physicians (*n* = 480)	Others (*n* = 193)	*p*-value
Attitude part				
1. Generic drugs evaluated through consistency can completely replace the original drugs.				0.001
Strongly disagree	21 (3.6%)	21 (4.4%)	8 (4.1)	
Disagree	138 (23.6%)	157 (32.7%)	59 (30.6)	
Neutral	204 (34.9%)	162 (33.8%)	42 (21.8)	
Agree	182 (31.2%)	115 (24.0%)	71 (36.8)	
Strongly agree	38 (6.7%)	25 (5.2%)	13 (6.7)	
2. National centralized procurement policy helps to regulate clinical drug utilization behavior.				<0.01
Strongly disagree	6 (1.0%)	17 (3.5%)	2 (1.0%)	
Disagree	30 (5.1%)	61 (12.7%)	98 (50.8%)	
Neutral	138 (23.6%)	140 (29.2%)	9 (4.7%)	
Agree	301 (51.5%)	199 (41.5%)	48 (24.9%)	
Strongly agree	109 (18.7%)	63 (13.1%)	36 (18.7%)	
3. National centralized procurement policy helps to reduce the medical burden on patients.				<0.01
Strongly disagree	5 (0.9%)	5 (1.0) %	1 (0.5%)	
Disagree	8 (1.4%)	31 (6.5%)	98 (50.8%)	
Neutral	56 (9.6%)	87 (18.1%)	2 (1.0%)	
Agree	288 (49.3%)	244 (50.8%)	37 (19.2%)	
Strongly agree	227 (38.9%)	113 (23.5%)	55 (28.5%)	
4. National centralized procurement policy helps to improve the efficiency of medical insurance funds.				<0.01
Strongly disagree	5 (0.9%)	12 (2.5%)	2 (1.0%)	
Disagree	14 (2.4%)	36 (7.5%)	103 (53.4%)	
Neutral	75 (12.8%)	113 (23.5%)	5 (2.6%)	
Agree	311 (53.3%)	234 (48.8%)	38 (19.7%)	
Strongly agree	179 (30.7%)	85 (17.7%)	45 (23.3%)	
5. Hospitals should take various measures to encourage the priority use of centrally purchased drugs.				<0.01
Strongly disagree	4 (0.7%)	9 (1.9%)	3 (1.6%)	
Disagree	10 (1.7%)	52 (10.8%)	94 (48.7%)	
Neutral	64 (11.0%)	133 (27.7%)	9 (4.7%)	
Agree	316 (54.1%)	198 (41.3%)	43 (22.3%)	
Strongly agree	190 (32.5%)	88 (18.3%)	44 (22.8%)	
Practice part				
1. Would you actively follow the update of the national drug centralized procurement catalog?				<0.01
Never	18 (3.1%)	55 (11.5%)	24 (12.4%)	
Occasionally	125 (21.4%)	175 (36.5%)	78 (40.4%)	
Sometimes	113 (19.3%)	128 (26.7%)	42 (21.8%)	
Often	199 (34.1%)	102 (21.3%)	40 (20.7%)	
Always	129 (22.1%)	20 (4.2%)	9 (4.7%)	
2. Would you proactively explain the national drug procurement policy to patients?				<0.01
Never	17 (2.9%)	51 (10.6%)	33 (17.1%)	
Occasionally	128 (21.9%)	153 (31.9%)	62 (32.1%)	
Sometimes	155 (26.5%)	138 (28.7%)	51 (26.4%)	
Often	198 (33.9%)	117 (24.4%)	38 (19.7%)	
Always	86 (14.7%)	21 (4.4%)	9 (4.7%)	
3. Do you or your family use the drugs listed in the centralized procurement catalog?				<0.01
Never	20 (3.4%)	63 (13.1%)	26 (13.5%)	
Occasionally	79 (13.5%)	122 (25.4%)	45 (23.3%)	
Sometimes	141 (24.1%)	155 (32.3%)	61 (31.6%)	
Often	270 (46.2%)	112 (23.3%)	50 (25.9%)	
Always	74 (12.7%)	28 (5.8%)	11 (5.7%)	

### 3.5 Multivariate logistic regression analysis

A multivariate logistic regression analysis was conducted to identify the factors associated with KAP levels regarding the NCDP policy. For knowledge levels, pharmacists demonstrated significantly higher knowledge compared to physicians (OR = 4.14, 95% CI: 3.07–5.58, *p* < 0.01). Intermediate and senior professional titles were also significantly associated with higher knowledge levels (OR = 1.63, 95% CI: 1.15–2.33, *p* < 0.05; OR = 1.81, 95% CI: 1.11–2.96, *p* < 0.05). Additionally, attending training was significantly associated with higher knowledge levels (OR = 2.75, 95% CI: 2.14–3.53, *p* < 0.01). Positive attitudes were significantly associated with 11–20 years of working experience (OR = 0.63, 95%CI: 0.41–0.97, *p* < 0.05), holding a master’s degree or above (OR = 1.58, 95%CI: 1.02–2.45, *p* < 0.05), being a pharmacist (OR = 2.29, 95%CI: 1.71–3.08, *p* < 0.01) and attending training (OR = 1.65, 95%CI: 1.29–2.12, *p* < 0.01). For practice levels, pharmacists again showed significantly higher practice levels than physicians (OR = 3.86, 95% CI: 2.80–5.32, *p* < 0.01). Practice levels were also significantly higher among those with 11–20 years of working experience (OR = 2.36, 95% CI: 1.27–4.41, *p* < 0.05) and over 20 years of working experience (OR = 2.82, 95% CI: 1.31–6.07, *p* < 0.05). Attending training was significantly associated with higher practice levels (OR = 2.96, 95% CI: 2.25–3.88, *p* < 0.01) (see Table 5).

**Table 5:** Univariable and multivariable analysis of associated factors of HCPs’ KAP toward NCDP policy.

Variables	Knowledge level			Attitude level			Practice level		
	B	OR (95%CI)	*p*-value	B	OR (95%CI)	*p*-value	B	OR (95%CI)	*p*-value
Content	−0.76		<0.05	−0.12		0.67	−3.34		<0.01
Age(years)									
<30			Ref						Ref
30–39	−0.28	0.76 (0.45–1.27)	0.29				0.36	1.43 (0.78–2.63)	0.25
40–49	−0.25	0.78 (0.40–1.53)	0.47				0.41	1.51 (0.72–3.17)	0.27
≥50	−0.12	0.88 (0.39–2.02)	0.77				0.51	1.66 (0.69–3.99)	0.25
years of working									
≤5			Ref			Ref			Ref
6–10	0.30	1.35 (0.83–2.19)	0.23	−0.33	0.72 (0.49–1.07)	0.11	0.44	1.55 (0.88–2.72)	0.13
11–20	0.26	1.29 (0.74–2.23)	0.37	−0.46	0.63 (0.41–0.97)	<0.05	0.86	2.36 (1.27–4.41)	<0.05
>20	0.08	1.08 (0.53–2.23)	0.83	−0.22	0.80 (0.48–1.33)	0.39	1.04	2.82 (1.31–6.07)	<0.05
Professional title									
Junior			Ref			Ref			Ref
Intermediate	0.49	1.63 (1.15–2.33)	<0.05	0.24	1.26 (0.89–1.78)	0.18	−0.002	0.99 (0.67–1.45)	0.99
Senior	0.59	1.81 (1.11–2.96)	<0.05	0.32	1.38 (0.87–2.19)	0.18	0.29	1.34 (0.81–2.24)	0.26
Education level									
Technical secondary school and junior college			Ref			Ref			Ref
Bachelor	0.49	0.61 (0.35–1.06)	0.08	0.02	1.02 (0.61–1.72)	0.94	0.12	1.13 (0.65–1.97)	0.67
Master or above	−0.38	0.68 (0.43–1.08)	0.10	0.46	1.58(1.02–2.45)	<0.05	0.22	1.25 (0.80–1.95)	0.33
Occupation									
Physician			Ref			Ref			Ref
Pharmacist	1.42	4.14 (3.07–5.58)	<0.01	0.83	2.29 (1.71–3.08)	<0.01	1.35	3.86 (2.80–5.32)	<0.01
Others	0.32	1.38 (0.92–2.06)	0.12	−0.91	0.40 (0.27–0.60)	<0.01	0.22	1.25 (0.77–2.02)	0.36
Hospital category									
Tertiary hospitals			Ref			Ref			Ref
Secondary hospitals	−0.09	0.92 (0.68–1.23)	0.56	0.10	1.11 (0.82–1.49)	0.50	0.19	1.21 (0.90–1.64)	0.21
Attending training									
No			Ref			Ref			Ref
Yes	1.01	2.75 (2.14–3.53)	<0.01	0.50	1.65 (1.29–2.12)	<0.01	1.08	2.96 (2.25–3.88)	<0.01

### 3.6 HCPs’ common concerns with NCDP policy

**Figure 1:**
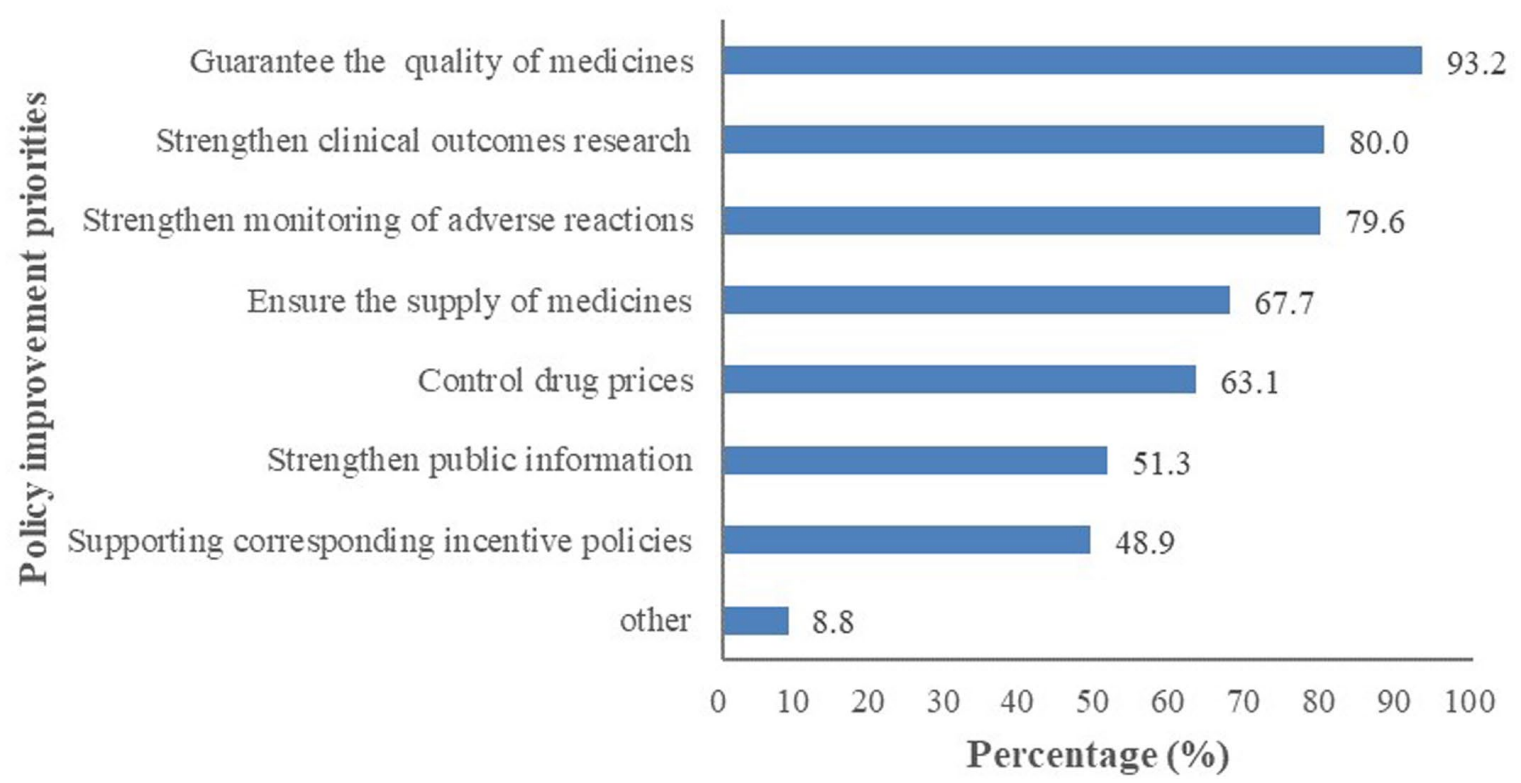
HCPs’ common concerns with NCDP policy.

The first three issues regarding the NCDP policy, according to HCPs, were guaranteeing the medicine quality (93.2%), strengthening clinical outcomes research (80.0%), and strengthening monitoring of adverse reactions (79.6%) (see Figure 1).

## 4 Discussion

The current study aimed to assess the KAP of HCPs regarding the NCDP policy in northwestern China. Compared to a previous study (20) conducted in central China, which found that only a small percentage of HCPs had good KAP toward the NCDP policy, our findings indicated that HCPs in Shaanxi have a better KAP level. Notably, this suggests varying levels of comprehension and involvement with the policy among participants.

To begin with, the average knowledge score suggested a moderate awareness among HCPs, indicating a general understanding but potentially lacking in-depth knowledge of the policy’s details. Only about half of the respondents agreed that the clinical efficacy and safety of original and generic drugs are identical. Similarly, many respondents acknowledged unfamiliarity with the identifier for drugs that have passed the GCE. As such, these findings suggested that HCPs have reservations about the quality of generic drugs. Recently, during the Shanghai “Two Sessions,” several medical experts have raised concerns that some centrally procured drugs may pose quality risks (30). The skepticism surrounding generic drugs stems from a lack of trust in the quality evaluation standards of these medications. This was mirrored in the survey findings, which indicated that only slightly over half of the respondents (55.8%) claimed to be aware of or had seen the consistency evaluation mark. Moreover, the substantially lower prices of generic drugs listed in the centralized procurement catalog, compared to their original counterparts, might contribute to this perception, leading some to equate price with quality.

Overall, attitudes toward the NCDP policy were generally positive, with the majority of respondents agreeing on its public benefit, signifying a recognition of the policy’s potential to enhance affordable medication access (31). However, the finding that only a small proportion strongly agreed that the NCDP policy helps to reduce the medical burden on patients indicated that there may be hesitations about the policy’s impact on patient welfare. The main reasons for this hesitation might be doubts about the quality of generic drugs, and some medical staff were also dissatisfied with the therapeutic effects of the centralized procurement of drugs (32). Although the GCE system has demonstrated pharmaceutical equivalence through *in vitro* pharmaceutical studies (e.g., dissolution profiles, bioavailability of active ingredients), the therapeutic equivalence in clinical practice requires further validation with real-world evidence. It should also be noted that current evaluation criteria primarily focus on critical quality attributes of active pharmaceutical ingredients (17) while potentially overlooking the impact of excipient variations in complex formulations (e.g., sustained-release dosage forms and inhalation preparations). Due to patent protections or trade secret restrictions on proprietary excipient compositions of originator drugs, generic manufacturers face challenges in achieving complete replication (33). Such discrepancies may lead to differences in release kinetics or local absorption efficiency, affecting therapeutic outcomes or safety profiles (34). Furthermore, the existing evaluation framework inadequately addresses systematic assessments of long-term stability, pharmacokinetic characteristics in special populations (e.g., patients with hepatic/renal impairment) (35), and rare adverse reactions. These limitations may contribute to HCPs’ reservations regarding the reliability of consistency-evaluated generic drugs. Our study revealed nuanced differences in attitudes toward the NCDP policy among various occupational groups of HCPs, particularly between pharmacists and physicians, underscoring profession-specific factors that sway policy acceptance. This pattern can be explained by role specialization; pharmacists, as medication supply chain managers, directly observe the impact of NCDP on drug accessibility and cost reduction. Higher education likely enhances their understanding of the policy’s design and long-term benefits. Additionally, workflow integration plays a key role, as pharmacists’ responsibilities in inventory management and shortage reporting align closely with NCDP objectives, fostering a sense of ownership and acceptance.

This finding also suggested that training effectively addresses the knowledge-attitude gap by bridging the critical information deficit physicians face in clinical decision-making, which relies heavily on drug efficacy and safety data, through providing evidence on generic equivalence and quality safeguards. Furthermore, training serves as a behavioral nudge by clarifying the availability of clinical flexibility, such as permitting the use of non-selected drugs under specific conditions, thereby reducing perceived constraints on clinical practice. These divergent patterns carry significant policy implications: pharmacists are well-positioned as NCDP advocates, and utilizing their expertise in supply chain coordination could enhance policy implementation. Meanwhile, physicians require targeted education to address therapeutic concerns, and mandatory training that incorporates real-world evidence, such as post-marketing studies of selected drugs, may bolster trust in the policy.

In terms of practicality, the mean score indicated that there is room for improvement in the implementation of the NCDP policy. The fact that only a minority of HCPs always or often actively follow the update of the NCDP catalog suggested a potential gap in staying informed about policy changes, which is crucial for effective policy implementation. Additionally, the finding that only about half of the HCPs would consider using centrally procured drugs for themselves or their families is disappointing, given the policy’s emphasis on affordability and accessibility. However, as the variety and coverage of these drugs continue to expand, we believe this proportion will gradually increase. Importantly, this result highlighted a significant gap in the consistent adoption of the policy across the board. The rapid frequency of updates in the centralized procurement policy poses difficulties for doctors in keeping track of policy changes in real-time (36). Work experience emerged as a significant factor influencing practice levels, while participation in training demonstrated a strong association with enhanced practice behaviors. These findings underscore the value of hands-on experience in policy implementation and highlight how targeted training can effectively bridge the gap between theoretical knowledge and practical application, thereby optimizing the execution of policy-related tasks.

The concerns of HCPs regarding the NCDP policy reflect the priorities and potential reservations, with the leading issues being guaranteeing medicine quality, strengthening clinical outcomes research, and strengthening monitoring of adverse reactions. These concerns are valid and must be addressed to ensure the successful implementation of the policy. Notably, future research should focus on several key areas. Firstly, high-quality clinical evidence is needed to evaluate the efficacy and safety of centrally procured drugs, especially in real-world settings. This will help to build confidence among HCPs and patients regarding the quality of these medications. Secondly, studies should explore the long-term impacts of the NCDP policy on patient outcomes and healthcare costs, providing a comprehensive assessment of its effectiveness. Thirdly, research should investigate the barriers to policy implementation from the perspectives of HCPs, patients, and policymakers and identify strategies to overcome these challenges. Additionally, the development of robust monitoring systems for adverse drug reactions is crucial to ensure patient safety and enhance the credibility of the policy. Similarly, targeted training is imperative to strengthen HCPs’ policy endorsement and implementation fidelity, with a priority focus on therapeutic effectiveness and safety evaluation. By clarifying bioequivalence certification methodologies (e.g., pharmacokinetic criteria) and quality assurance mechanisms, such education directly addresses the clinical concerns that hinder adherence to drug substitution, thereby turning evidence gaps into actionable competence.

## 5 Strengths and limitations

This study benefits from a large, diverse sample across multiple healthcare settings, enhancing its generalizability within similar contexts. The rigorous questionnaire validation and high response rate strengthen this study’s reliability. However, this study also had several limitations. Firstly, the limitations include its cross-sectional design, which precludes causal inferences, and potential selection bias due to convenience and snowball sampling. Secondly, self-reported data may also introduce social desirability bias, particularly in attitude and practice assessments. Thirdly and importantly, the study participants were exclusively drawn from HCPs in public hospitals, excluding those from primary healthcare institutions. Future research should focus on primary healthcare institutions, as they play a vital role in the healthcare system and provide unique insights not captured in the current study. Additionally, the focus on Shaanxi Province—a region with higher GDP and healthcare infrastructure—may limit extrapolation to less-developed areas in China. Also, future studies with larger samples should explore finer subgroup stratifications (e.g., by pharmacy type, institutional context) to uncover additional determinants.

## 6 Conclusion

This study investigated healthcare professionals’ KAP regarding China’s NCDP policy in Northwest China. Key findings revealed moderate but suboptimal overall KAP levels, with only 58.4% demonstrating adequate knowledge, 63.2% holding positive attitudes, and 35.2% exhibiting good practices. Pharmacists significantly outperformed physicians across all KAP domains due to their specialized drug management training and policy-aligned roles, while training attendance consistently predicted enhanced KAP across occupational groups. Despite the general acknowledgment of the policy’s public health benefits, concerns about generic drug quality and therapeutic equivalence, particularly regarding clinical efficacy and safety, have persistently emerged as central barriers to its implementation. Significant KAP variations across professions, education levels, and work experience underscore the need for tailored interventions: evidence-based medicine training for physicians to address therapeutic equivalence concerns, drug management protocols for nurses, and targeted educational campaigns reinforcing generic drug quality assurance. To optimize the integration of NCDP policies into healthcare systems, strengthening real-world evidence generation and implementing profession-specific strategies are crucial. Tailored strategies can enhance the effectiveness of NCDP policies and increase their acceptance among healthcare professionals, thereby ensuring the policies’ successful integration.

## Data Availability

The original contributions presented in the study are included in the article/Supplementary material, further inquiries can be directed to the corresponding author.

## Footnotes

^1^ https://www.calculator.net/sample-size-calculator.html
