# Supplementary material for: Healthcare professionals’ knowledge, attitudes, and practices regarding China’s National Centralized Drug Procurement policy: a cross-sectional study in Shaanxi Province
Source: Front Public Health. 2025 Aug 20;13:1616534. doi: 10.3389/fpubh.2025.1616534 (PMC12405221; doi:10.3389/fpubh.2025.1616534)
Supplement: Supplementary file 1 [file Data_Sheet_1.pdf]

Questionnaire on Healthcare Professionals' Knowledge, Attitudes, and Practices (KAP) Regarding China's National Centralized Drug Procurement Policy

Instructions:

This questionnaire aims to understand healthcare professionals' awareness and perspectives on China's National Centralized National Centralized Drug Procurement Policy (also known as "Volume-Based Procurement").Generic Consistency Evaluation (GCE) refers to the scientific assessment process to confirm that generic drugs are equivalent to their original branded counterparts in quality, safety, and efficacy. Please answer based on your actual situation. All data are anonymous and for research purposes only. Thank you!

1. Gender

   □ Male □ Female

2. Age (years):

   □<30 □30-39 □40-49 □≥50

3. Years of work experience:

   □≤5 □6-10 □11-20 □＞20

4. Professional title

   □ Senior □ Intermediate □ Junior/Uncertified

5. Education level

   □ Doctoral degree □ Master's degree

   □ Bachelor's degree □ Technical secondary school and junior college

6. What region are you from? ______

7. Occupation

   □ Physician □ Pharmacist □ Others

8. Hospital category

   □ Tertiary hospitals □Secondary hospitals

9. Are you from a public hospital ?

   □ Yes □ No

10. Have you ever attended any training related to centralized procurement policies?

    □ Yes □ No

11. How familiar are you with China's NCDP Policy?

☐ Very familiar □ Relatively familiar

☐ Moderately familiar □ Slightly familiar

☐ Never heard of it

12. Do you know that China has fully carried out consistency evaluation of generic drugs？

□ Yes □ No

13. Do you know the relationship between centralized volume procurement and consistency evaluation？

□ Yes □ No

14. Do you know that not all drugs included in the centralized procurement catalog are generic drugs？

□ Yes □ No

15. Do you know/have you seen the identification of drugs that have passed the consistency evaluation？

□ Yes □ No

16. Do you think the efficacy of generic drugs evaluated through consistency is the same as that of original drugs?

□ Yes □ No

17. Do you think the safety of generic drugs evaluated through consistency is the same as that of original drugs?

□ Yes □ No

18. Do you agree that NDCP policy can alleviate the payment pressure of medical insurance funds？

□ Yes □ No

19. Which of the following ways do you mainly use to obtain information on medicine centralized procurement policies?

□Workplace training □Colleagues □ Family and friends

□ Online platforms (websites, WeChat public accounts, Douyin, etc.)

□ Others

20. What’s your attitude to the national centralized medicine procurement?

□ It’s all advantages

□ Advantages outweigh disadvantages

□ Not sure

□ Disadvantages outweigh advantages

□It’s all disadvantages

21. Generic drugs evaluated through consistency can completely replace the original drugs.

□ Strongly disagree □ Disagree □ Neutral

□ Agree □ Strongly agree

22. National centralized procurement policy helps to regulate clinical drug utilization behavior.

□ Strongly disagree □ Disagree □ Neutral

□ Agree □ Strongly agree

23. National centralized procurement policy helps to reduce the medical burden on patients

□ Strongly disagree □ Disagree □ Neutral

□ Agree □ Strongly agree

24. National centralized procurement policy helps to improve the efficiency of medical insurance funds.

□ Strongly disagree □ Disagree □ Neutral

□ Agree □ Strongly agree

25. Hospitals should take various measures to encourage the priority use of centrally purchased drugs.

□ Strongly disagree □ Disagree □ Neutral

□ Agree □ Strongly agree

26. Would you actively follow the update of the national drug centralized procurement catalog?

□ Never □ Occasionally □ Sometimes □ Often □ Always

27. Would you proactively explain the national drug procurement policy to patients?

□ Never □ Occasionally □ Sometimes □ Often □ Always

28. Do you or your family use the drugs listed in the centralized procurement catalog?

    □ Never □ Occasionally □ Sometimes □ Often □ Always

29. Do you prioritize medicine from centralized procurement when prescribing? (For doctors)

    □ Never □ Occasionally □ Sometimes □ Often □ Always

30. What’s your main reason for prescribing centrally procured medicines? (For doctors)

    □Recognizing their clinical efficacy

    □Departmental target tasks

    □Hospital only stocks these, with no alternatives

    □ Lower price, less financial burden on patients

    □ Patient request □ Can’t tell if it’s procured

31. Always Would you proactively recommend procured medicines if patients ask about them? (For pharmacists)

    □ Never □ Occasionally □ Sometimes □ Often □ Always

32. Regarding the implementation of the national medicine centralized procurement policy, which of the following aspects do you think should be the most important to focus on?

    □ Ensuring medicine quality □ Conducting clinical efficacy studies

    □ Monitoring adverse reactions □ Controlling medicine prices

    □ Ensuring supply guarantees □ Supporting incentive policies

    □ Public education campaigns □ Others (Please specify)

33. Based on your experience, what aspects of the current centralized procurement policy do you think need improvement? (Please specify) ____________________
